# An exploration of job stress and health in the Norwegian police service: a cross sectional study

**DOI:** 10.1186/1745-6673-1-26

**Published:** 2006-12-11

**Authors:** Anne Marie Berg, Erlend Hem, Bjørn Lau, Øivind Ekeberg

**Affiliations:** 1 Department of Behavioural Sciences in Medicine, Institute of Basic Medical Sciences, Faculty of Medicine, University of Oslo, PO. Box 1111 Blindern, NO-0317 Oslo, Norway; 2 National Institute of Occupational Health, Pb. 8149 Dep, NO-0033 Oslo, Norway

## Abstract

**Background:**

Police work is regarded as a high-stress occupation, but so far, no nationwide study has explored the associations between work stress and health.

**Aims:**

To explore physical and mental health among Norwegian police and associations to job stress. Comparisons were made with a nationwide sample of Norwegian physicians and the general Norwegian population.

**Methods:**

Comprehensive nationwide questionnaire survey of 3,272 Norwegian police at all hierarchical levels, including the Norwegian Police Stress Survey with two factors (serious operational tasks and work injuries), the Job Stress Survey with two factors (job pressure and lack of support), the Basic Character Inventory, the Subjective Health Complaint questionnaire, the Hospital Anxiety and Depression Scale, the Maslach Burnout Inventory, and Paykel's Suicidal Feelings in the General Population.

**Results:**

The frequency of job pressure and lack of support was mainly associated to physical and mental health problems. Females showed higher means on anxiety symptoms than males (4.2, SD 2.9 and 3.7, SD 2.9, respectively; p < 0.01), while males showed higher means on depressive symptoms (3.1, SD 2.9 and 2.4, SD 2.5, respectively; p < 0.001). Police reported more subjective health complaints, depersonalization and higher scores on three of four personality traits than physicians, but lower scores on anxiety and depressive symptoms than the general population.

**Conclusion:**

This is the first nationwide study to explore job stress and physical and mental health in police. The results indicate that Norwegian police have high levels of musculoskeletal health problems mainly associated to the frequency of job pressure and lack of support. However, also frequent exposure to work injuries was associated to health problems. This may indicate that daily routine work as well as police operational duties must be taken into consideration in assessing job stress and police health.

## Background

Police work has often been regarded as a stressful occupation; in fact, it has been described as one of the most stressful occupations in the world [1]. However, previous studies have found that police work is not a particularly stressful occupation, but may be a factor of psychological distress [2,3], and that police stress is not characteristically different from stress in some other occupations [3,4]. However, routine occupational stress may be a factor of psychological distress [5].

The physical threats in police operational duties have been regarded as inherent causes of stress in police work, but organizational factors such as work overload, time pressure, inadequate resources, manpower shortage, lack of communication, managerial styles etc. emerge as more stressful [6-8]. This may indicate that police are trained for police operational duties [2], whereas their ability to cope with organizational stressors may be less adequate.

The negative impact of stress in police work is manifested in different ways, such as somatic and mental health problems and burnout [3,4,7,8], and it depends on the frequency, the intensity and how the experienced situation is perceived [9,10]. Data on frequency is important in determining which stressors have had the greatest impact on daily police work [11].

Previous research has emphasized individual differences when it comes to stress and work. Here, the focus of interest has been in personality factors. Two prominent concepts have been locus of control and neuroticism [12]. Neuroticism tends to correlate with psychological distress [2] and is an independent predictor of burnout in police [10]. Attitudes and behavioural characteristics generated by police work itself can lead to rigidity, suspiciousness, cynicism and authoritarianism, which are attributed to burnout [13]. There are large variations in police work between countries and even within the same country. These features suggest that more information on the different aspects of police work that cause stress and police-specific measures are needed from nationwide and comparative studies [14]. During the last few years, human service occupations have been extensively studied. For example, burnout may occur particularly often among individuals who work in the human service professions. Recently, a doctoral thesis studied suicidal behaviour among human service occupations in Norway especially among physicians and police [15]. The study showed a significantly higher level of suicidal thoughts, attempts and suicide among doctors than police.

Police officers in Norway are well educated and a selected group. The selection criteria for admission to three years of study at the Police Academy is completion of high school and physical and mental tests. During the years at the Academy, the recruits are trained thoroughly in specific tasks, a process which is intended to prepare them for operational duties. The requirements of good health and proper training to be a police officer are unquestionably very important. However, this "perfect" image that starts already at the Police Academy may also constitute a disadvantage to police employees in that it may encourage a general attitude towards the police that they do not have work related or personal problems, especially not mental health problems. A consequence of this attitude may be that police underreport symptoms, especially mental health symptoms. There has, however, never been conducted a large scale study trying to explore the relationship between working conditions and health in Norwegian police. The present paper is part of the first comprehensive, nationwide, cross-sectional study to attempt to gather knowledge about some of these issues in the police service. Three previous articles on the basis of the present cohort have been published so far [16-18], but there is no overlap between the data presented in this paper and the previous published articles.

The aims of the study were:

1 To explore physical and mental health in the Norwegian police service.

2 To explore the relationship between the frequency and severity of perceived job stress and health problems.

3 To compare health problems in the Norwegian police service with a representative sample of Norwegian physicians on subjective health complaints, personality traits and burnout, in addition to anxiety and depressive symptoms in the general Norwegian population.

## Methods

Participants in this study included officers, middle managers and managers. Hence, the term 'police' is used to describe respondents in the general sample. Policing in Norway comprises three categories: Investigation, Uniformed policing, and Administration. They were all members of the largest police industrial organization in Norway, The Norwegian Police Union, of which approximately 95% of the police service are voluntary members. The police service in Norway comprises two types of districts: urban districts and rural ('lensman') districts. The two categories have the same education and training, but in the rural districts they work in smaller communities, often including large country areas with scattered houses. The number of police is typically small. Urban districts serve larger communities and cities. The term 'inhabitants' in the study is used to describe the people who reside and/or work in the districts. The sample is described in detail elsewhere [16]. The project was approved by the Norwegian Data Inspectorate and the Regional Committee for Research Ethics.

Some results were compared with the Norwegian Physicians' Survey; a large-scale nationwide study conducted in 1993 [19]. Police and physicians share some similarities, as they are both human service occupations, and they may be exposed to high stress. The study included active members of the Norwegian Medical Association (25 to 70 years). Data were collected by means of overlapping questionnaires. Out of 16 different questionnaires, each physician received one primary questionnaire (response rate 71.8%, N = 6,652) and three randomly selected secondary ones [18]. In the present study, comparisons are made on subjective health complaints from the primary questionnaire, whereas personality traits (response 896 physicians, 72.9%) and burnout (response 1,082 physicians, 73.3%) were from the additional questionnaires.

Comparisons with respect to anxiety and depressive symptoms were made with the Nord-Trøndelag Health Study (HUNT), comprising a large representative sample of the general population in Norway. In the HUNT study 61,216 persons had valid responses on the HADS (The Hospital Anxiety and Depression Scale) dimensions out of 92,100 eligible [20,21]. Totally, 65,648 (71.3%) participated in the HUNT study [20]. The police sample was compared with the age group from 20 to 59 years.

### Distribution of the questionnaire

In December 2000, a questionnaire was distributed by The Norwegian Police Union to presumably all 6,398 police educated members. The questionnaire included 396 questions on background information, physical and mental health, working conditions, job satisfaction, burnout, coping, personality and suicidal ideation. Respondents were anonymous and the instrument was distributed once. Several written reminders were distributed through trade union representatives and the internal data system of the police service. The final response rate was 51%, which represents a total of 3,272 persons. The sample is presented in Table 1.

**Table 1:** Description of the police sample

		Frequency	Per cent	Per cent total police population in Norway	Significance
Gender	Women	501	15.7	17.9	χ^2 ^= 4.6 *
	Men	2,692	84.3		
Age (years)	Total sample (102 did not answer)	3,170			
	20–29	509	16.1		
	30–39	1,175	37.1		
	40–49	1,047	33.0		
	50–59	430	13.6		
Marital status	Single	342	10.6		
	Married/common law	2,715	84.3		
	Separated/divorced	164	5.1		
Rank	Upper management	96	2.9	9.6	χ^2 ^= 144.3***
	Middle management	1,034	31.7	32.3	χ^2 ^= ns
	Non-management	2,128	65.3	58.1	χ^2 ^= 49.3***
Service	Rural police districts	870	26.6	23.0	χ^2 ^= 24.3 ***
	Urban police districts	2,399	73.4	77.0	
Main task	Investigation	1,379	43.4		
	Uniformed policing	1,286	40.5		
	Administration	513	16.1		
Inhabitants	> 50,000	1,626	51.2		
	20,000 – 50,000	648	20.4		
	5,000 – 20,000	728	22.9		
	< 5,000	175	5.5		

Note. *p < 0.05, ***p < 0.001.

The sample was not representative of the total police population, i.e. the present sample was younger (38.9 vs. 40.2 years; t = 8.3, p < 0.001), women and upper management were underrepresented, whereas non-management and rural police were overrepresented. However, the sample was representative compared to all members of the Police Union.

Due to problems in distributing the questionnaire, as described previously [16], 680 letters were distributed to randomly selected police from the original sample in November 2001, asking whether they had received the questionnaire or not. The response rate was 70% (n = 475). The results showed that 26% had never received the questionnaire. Based on this figure, the true response rate is higher than 51%.

### The Job Stress Survey

The Job Stress Survey (JSS) [22] is designed to determine which conditions in the workplace cause stress. The JSS consists of 30 items that describe work-related events and situations ('stressors') encountered in a wide variety of occupations. The 30 stressors are assessed on a nine-point perceived-severity rating scale from 0 to 9+, on severity and frequency during the last six months.

Twenty JSS items constitute the two main factors: (1). Job pressure, including ten items mainly related to organizational work and (2). Lack of support, including ten items related to working environment and leadership. These factors can then be analysed on three different levels: severity, frequency, and a severity*frequency index. Cronbach's alphas for the severity and frequency of job pressure were 0.83 and 0.85, respectively, whereas Cronbach's alphas for the severity and frequency of lack of support were 0.83 and 0.85, respectively.

### The Norwegian Police Stress Survey

The Norwegian Police Stress Survey (NPSS) was developed for the present study using the 60-item Police Stress Survey [23] as a starting point. Sixteen items were drawn from the Police Stress Survey, of which ten were unaltered and six were modified to be relevant to Norwegian police work. An example of such a modified question is: 'Fellow police killed in the line of duty' to 'Fellow police hurt in the line of duty'. Based on interviews with some Norwegian police in various positions, 20 additional questions were developed especially for Norwegian conditions. An example of these items is 'Take care of individuals with mental illness.'

To identify a factor structure in these 36 items, we conducted principal component analyses with promax rotation. To be included in the structure, an item had to load on the same factor with respect to both severity and frequency. Based on this procedure, 10 items specific for police work were identified and included in the NPSS: (1) serious operational tasks, which included six items related to operational daily police work; and (2) work injuries, which included four items related to damage or accidents toward members of the public, peers or respondents themselves during police work. Cronbach's alphas for the severity and frequency of serious operational tasks were 0.82 and 0.83, respectively. Cronbach's alphas for the severity and frequency of work injuries were 0.84 and 0.76, respectively.

### Personality

The personality inventory used in this study was the Basic Character Inventory [24,25]. This instrument contains 36 items and is based on the 'Big three' personality dimensions of neuroticism (for example, 'I'm very touchy about criticism'), extroversion (for example, 'Many people consider me a lively person'), control/compulsiveness (for example, 'Everything I do must be precise and accurate'), with an additional fourth dimension called reality weakness (for example, 'I experience myself as being totally different at different points in time'). Each dimension is based on nine questions with responses on a Likert scale between 0 (low) and 9 (high).

### Subjective Health Complaints

The subjective experience of health was assessed by a ten-item version of the Subjective Health Complaint (SHC) questionnaire. This questionnaire consists of questions examining the occurrence, intensity and duration of muscle/skeleton pain, migraine/headache, and digestive problems for the last 30 days [26,27]. Seven of the 10 items are related to musculoskeletal symptoms. The items are scored on a four-point scale ranging from no complaints (0) to serious complaints (3). In the present study, the SHC sum score was transformed to a dichotomous variable. Consistent with a previous study [16], those who had a response of 2 or 3 on at least one of the ten items were scored as 'cases'. No diagnosis was given.

### Anxiety and depressive symptoms

The Hospital Anxiety and Depression Scale (HADS) [28] included 14 questions, divided into an anxiety and a depression subscale. Each subscale contained seven items and was scored on a four-point scale. In the present study, the two subscales were used as both continuous and dichotomized variables, with cut-off scores for both subscales of 8+ [29].

### Burnout

Burnout was measured with a 22-item version of the Maslach Burnout Inventory (30). The inventory contains questions regarding three factors that specify burnout: emotional exhaustion (MBI-A), depersonalization/cynicism (MBI-B) and personal accomplishment (MBI-C). The items are scored on a five-point scale. In the present study, the MBI sum scores were dichotomized at the 50^th ^percentile.

### Suicidal behaviour

The prevalence of suicidal ideation and attempts was assessed by a modified questionnaire, originally introduced by Paykel et al. [31]. Paykel's Suicidal Feelings in the General Population questionnaire contains five questions, of which one question was used in the present study: 'Have you ever reached the point where you seriously considered taking your life, or perhaps made plans how you would go about doing it?' This question contained six response possibilities: never, once, 2–3 times, 4–5 times, 6–9 times and at least 10 times. The response to the question was dichotomized into never (0) and any frequency (1) prior to statistical analyses.

### Self reported health

Overall health was measured by one question; "In general, how do you rate your health?" to which responses were on a five-point scale: "Very good", "Good", "Neither good nor bad", "Bad" and "Very bad".

### Statistical analyses

χ^2 ^tests were used to measure the differences between the study sample and the total police population according to gender, rank and service. Student's t-test was used to test the differences between the sample and the total police population according to age. Unianova (F-test) was used to test differences on means between the police and the physicians. To test whether the police sample differed from the general population on anxiety and depressive symptoms, we used a One-Sample t-test where the mean values from the general population were specified as constants. In order to test whether the stress factors were able to predict cases of anxiety and depressive symptoms, somatic health complaints, burnout or serious suicidal ideation, a series with logistic regression analysis were conducted. Age, gender and personality were controlled for, in addition to the health variables.

## Results

Self reported overall health is good in Norwegian police: 88.3% of respondents (females 90.2%; males 88.1%; NS) reported that they considered their health as very good or good. Good health declined with age in both genders, more among women than men.

Table 2 shows descriptive statistics and gender differences on all health variables. Even though the differences according to gender were generally highly statistically significant, the crude differences were rather small. Males reported more burnout and depressive symptoms, but had lower anxiety scores than females. Females had higher scores on all personality traits, particularly on neuroticism (3.56 vs. 2.34; p < 0.001).

**Table 2:** Descriptive statistics and gender differences for burnout, health, personality, and work stress

	**Females**		**Males**		
	mean	SD	mean	SD	p
**Burnout**					
Emotional exhaustion (MBI)^a^	2.14	0.64	2.25	0.70	**
Depersonalization (MBI)^a^	2.12	0.68	2.26	0.76	***
Personal accomplishment (MBI)^a^	2.48	0.42	2.42	0.41	**
					
**Health**					
Anxiety subscale (HADS)^b^	4.2	2.9	3.7	2.9	**
Depression subscale (HADS)^b^	2.4	2.5	3.1	2.9	***
Subjective Health Complaint	4.27	3.84	3.87	4.3	*
					
**Personality**					
Neuroticism (BCI)^c^	3.56	2.26	2.34	2.03	***
Extroversion (BCI)^c^	5.91	2.28	5.11	2.4	***
Control/compulsiveness (BCI)^c^	4.46	2.2	4.32	2.12	Ns
Reality weakness (BCI)^c^	1.38	1.7	1.19	1.51	*
					
**Job stress**					
*Severity*					
Job Pressure	4.8	1.0	4.7	1.1	Ns
Lack of Support	5.4	1.2	5.2	1.2	*
Serious Operational Tasks	5.7	1.2	5.5	1.2	***
Work Injuries	6.8	1.4	6.3	1.5	***
*Frequency*					
Job Pressure	3.8	2.3	4.2	2.2	***
Lack of Support	1.7	1.5	2.1	1.7	***
Serious Operational Tasks	2.5	2.0	2.7	2.1	*
Work Injuries	0.2	0.4	0.4	0.7	***

Note. *p < 0.05, **p < 0.01, ***p < 0.001.

^a ^MBI – Maslach Burnout Inventory

^b ^HADS – Hospital Anxiety and Depression Scale

^c ^BCI – Basic Character Inventory

The frequency of job pressure was high (4.1), while the frequency of work injuries was low (0.3), with the others in between. The opposite pattern was shown for severity, as work injuries had the highest mean score (6.3) and job pressure had the lowest (4.8). Women perceived the stressors to be less frequent but more severe than men.

Table 3 shows that police had higher mean scores than physicians on subjective health complaints (women 4.27 vs. 2.76; p < 0.001, men 3.87 vs. 2.00; p < 0.001). A total of 40.7% of the police (females 46.2%; males 39.7%; p = 0.007) reported subjective health complaint cases, which is significantly more than the 29.6% among Norwegian physicians (females 32.3%; males 27.9%).

**Table 3:** Group differences between police and Norwegian physicians. Physicians: Subjective health complaints (N = 6,652); Personality (N = 896); Burnout (N = 1,082)

		**Police**		**Physicians**		
**Females**		mean	SD	mean	SD	p
	Subjective Health Complaints	4.27	(3.84)	2.76	(2.89)	***
	(BCI)^c ^neuroticism	3.56	(2.26)	4.00	(2.22)	*
	(BCI)^c ^extroversion	5.91	(2.28)	5.51	(2.66)	*
	(BCI)^c ^control/compulsiveness	4.46	(2.20)	3.37	(2.14)	***
	(BCI)^c ^reality weakness	1.38	(1.70)	0.98	(1.40)	**
	(MBI)^a ^emotional exhaustion	2.15	(0.64)	2.64	(0.86)	***
	(MBI)^a ^depersonalization	2.13	(0.68)	1.80	(0.60)	***
	(MBI)^a ^personal accomplishment	2.48	(0.42)	2.52	(0.46)	*
						
**Males**						
	Subjective Health Complaints	3.87	(4.30)	2.00	(2.32)	***
	(BCI)^c ^neuroticism	2.34	(2.03)	2.83	(2.10)	***
	(BCI)^c ^extroversion	5.11	(2.40)	4.89	(2.50)	*
	(BCI)^c ^control/compulsiveness	4.32	(2.12)	3.51	(2.05)	***
	(BCI)^c ^reality weakness	1.19	(1.52)	0.91	(1.23)	***
	(MBI)^a ^emotional exhaustion	2.25	(0.69)	2.56	(0.81)	***
	(MBI)^a ^depersonalization	2.26	(0.76)	1.88	(0.64)	***
	(MBI)^a ^personal accomplishment	2.42	(0.41)	2.41	(0.44)	Ns

Note. *p < 0.05, **p < 0.01, ***p < 0.001.

^a ^MBI – Maslach Burnout Inventory

^c ^BCI – Basic Character Inventory

Physicians had significantly higher mean scores on emotional exhaustion than police (women 2.64 vs. 2.15; p < 0.001, men 2.56 vs. 2.25; p < 0.001), while police had higher mean scores on depersonalization (women 2.13 vs.1.80; p < 0.001, men 2.26 vs. 1.88; p < 0.001). In general, the younger age groups of both genders in police reported lower levels of anxiety and depressive symptoms than the corresponding general population (see Table 4).

**Table 4:** Group differences between police and a general Norwegian population sample. General population: Hospital Anxiety and Depression Scale (N = 61,216)

		**Police**		**General population**		
**Females**		mean	SD	mean	SD	p
Age	HADS-A^b^					
20–29		4.0	(2.6)	4.5	(3.2)	*
30–39		4.2	(3.0)	4.6	(3.4)	*
40–49		4.2	(3.3)	4.6	(3.5)	Ns
50–59		4.1	(3.2)	4.8	(3.6)	Ns
	HADS-D^b^					
20–29		1.6	(1.9)	2.2	(2.4)	***
30–39		2.5	(2.5)	2.7	(2.8)	Ns
40–49		3.0	(3.1)	3.2	(3.0)	Ns
50–59		3.2	(3.2)	3.7	(3.1)	Ns
**Males**						
Age	HADS-A^b^					
20–29		3.2	(2.3)	4.1	(2.9)	***
30–39		3.7	(2.8)	4.2	(3.1)	***
40–49		4.0	(3.1)	4.2	(3.3)	*
50–59		3.6	(3.3)	4.0	(3.6)	*
	HADS-D^b^					
20–29		1.5	(1.9)	2.4	(2.4)	***
30–39		2.8	(2.8)	2.9	(2.7)	Ns
40–49		3.6	(3.1)	3.6	(3.0)	Ns
50–59		3.7	(3.2)	4.1	(3.2)	*

Note. *p < 0.05, ***p < 0.001.

^b ^HADS – Hospital Anxiety and Depression Scale

The association between health problems and burnout was measured by adjusted logistic regression analysis (see Table 5). The frequency of job pressure was independently associated with anxiety symptoms (OR 1.6, 95% CI = 1.2–2.1), subjective health complaints (OR 1.4, 95% CI = 1.2–1.7) and the three burnout dimensions. The severity of job pressure was associated with anxiety symptoms (OR 2.0, 95% CI = 1.5–2.7) and two burnout dimensions.

**Table 5:** Associations between physical and mental health in police. Adjusted model controlled for age, gender, personality, and the other health variables.

Predictors				
	Job Pressure – Frequency	Lack of Support – Frequency	Serious Operational Tasks – Frequency	Work Injuries – Frequency
	OR (95%CI)	OR (95%CI)	OR (95%CI)	OR (95%CI)
(HADS)^b ^Anxiety	1.6** (1.2 – 2.1)	1.5** (1.1 – 2.1)	0.9 (0.7 – 1.2)	1.0 (0.7 – 1.3)
(HADS)^b ^Depression	0.9 (0.6 – 1.2)	1.5* (1.1 – 2.2)	1.0 (0.7 – 1.5)	1.4* (1.0 – 1.9)
Subjective Health Complaints	1.4*** (1.2 – 1.7)	1.4*** (1.2 – 1.7)	1.0 (0.8 – 1.2)	1.2* (1.0 – 1.4)
(MBI)^a ^emotional exhaustion	1.9*** (1.6 – 2.2)	2.0*** (1.7 – 2.7)	1.3** (1.0 – 1.5)	1.1 (0.9 – 1.4)
(MBI)^a ^depersonalization	1.3** (1.1 – 1.5)	1.3** (1.1 – 1.5)	1.8*** (1.5 – 2.2)	1.3** (1.1 – 1.6)
(MBI)^a ^personal accomplishment	0.6*** (0.6 – 0.7)	0.8* (0.7 – 1.0)	0.7*** (0.6 – 0.8)	0.7*** (0.7 – 0.9)
Suicidal ideation	1.1 (0.8 – 1.6)	1.4 (1.0 – 2.0)	1.0 (0.7 – 1.4)	1.0 (0.7 – 1.4)

	Job Pressure – Severity	Lack of Support – Severity	Serious Operational Tasks – Severity	Work Injuries – Severity

	OR (95%CI)	OR (95%CI)	OR (95%CI)	OR (95%CI)
(HADS)^b ^Anxiety	2.0*** (1.5 – 2.7)	1.2 (0.9 – 1.7)	1.7*** (1.2 – 2.3)	1.0 (0.8 – 1.4)
(HADS)^b ^Depression	1.0 (0.7 – 1.4)	1.3 (0.9 – 1.99	0.8 (0.5 – 1.1)	1.1 (0.8 – 1.5)
Subjective Health Complaints	1.1 (1.0 – 1.3)	1.4*** (1.2 – 1.7)	1.0 (0.9 – 1.2)	0.9 (0.8 – 1.1)
(MBI)^a ^emotional exhaustion	2.1*** (1.8 – 2.5)	1.8*** (1.5 – 2.2)	1.3** (1.1 – 1.6)	1.4*** (1.1 – 1.6)
(MBI)^a ^depersonalization	0.9 (0.8 – 1.1)	0.9 (0.8 – 1.1)	1.0 (0.9 – 1.2)	0.9 (0.8 – 1.1)
(MBI)^a ^personal accomplishment	1.3*** (1.1 – 1.6)	1.1 (0.9 – 1.2)	1.6*** (1.3 – 1.8)	1.1 (0.9 – 1.3)
Suicidal ideation	0.8 (0.6 – 1.19	1.3 (0.9 – 1.7)	1.2 (0.9 – 1.7)	1.3 (0.9 – 1.7)

Note. *p < 0.05, **p < 0.01, ***p < 0.001.

^a ^MBI – Maslach Burnout Inventory

^b ^HADS – Hospital Anxiety and Depression Scale

The frequency of lack of support was associated with anxiety and depressive symptoms (both OR 1.5, 95% CI = 1.1–2.1 and 1.1–2.2, respectively), subjective health complaints (OR 1.4, 95% CI = 1.2–1.7) and the three burnout dimensions. The severity of lack of support was only associated with subjective health complaints and one burnout dimension.

The frequency of serious operational tasks was associated with the three burnout dimensions. The severity of serious operational tasks was associated with anxiety symptoms (OR 1.7, 95% CI = 1.2–2.3) and two burnout dimensions.

The frequency of work injuries was associated with depressive symptoms (OR 1.4, 95% CI = 1.0–1.9), subjective health complaints (OR 1.2, 95% CI = 1.0–1.4) and two burnout dimensions, whereas severity of work injuries only was associated with the burnout dimension emotional exhaustion (OR 1.4, 95% CI = 1.1–1.6).

## Discussion

Self reported physical health was reported to be generally good and to decrease by age, which is in accordance with findings in the general population [32].

About 40% were "cases" according to subjective health complaints, which was significantly higher than among physicians. Females in both occupations reported significantly more subjective health complaints than males. Studies have shown "cases" between 23%–40% in police measured by the General Health Questionnaire, which is reported to be higher than in the general population, but equal or lower than other occupational groups such as civil servants and teachers [3,9]. In the present study, however, seven of the ten items of somatic health complaints comprised of musculoskeletal symptoms. The original SHC scale contains 29 items on a wider range of subjective health complaints. Based on the fact that police in Norway are a selected group regarding physical and mental health, the "cases" on subjective health complaints may seem surprising. However, this may indicate a rather high level of tensions at work that are converted to bodily symptoms, which may cover emotional distress. The lower levels of anxiety and depressive symptoms among the youngest age groups in police compared with the general population may indicate that younger police have better mental health than the general population, which may be related to the selection process, but a cohort or age effect or report bias may also be relevant.

Gender differences were shown on nearly all variables on health and stress factors. Males reported overall more burnout than females, while females reported significantly more neuroticism, extroversion and reality weakness than males. Female police perceived all stress factors more severely than males, although they experienced all factors less frequently. Police work may have different impacts on males and females. Women may feel more isolated and undervalued by colleagues and experience greater ambivalence from the public towards them as police [8]. Although the proportion of female police is increasing in Norway, which is a highly liberated country, there is still a need for further studies of gender issues in policing.

The frequency of job pressure and lack of support was associated with more subjective health problems and anxiety symptoms than serious operational tasks and work injuries. However, frequent exposure to work injuries was associated to somatic health complaints and the frequency of lack of support and work injuries was associated to depressive symptoms. This indicates that both daily hassles and police operational duties should be taken into consideration when it comes to assessing impacts on police health. Daily hassles may even be of special importance, as police officers are trained to cope with serious operational duties. The experience of not coping well may result in distress and health problems. All stress factors were associated with burnout in police. Interestingly, the frequency, but not the severity, of stress factors was associated with depersonalization (cynicism). Too much job stress in police may contribute to a breakdown in adaptation that results from the long-term imbalance of demands and resources [33] and may result in cynicism.

### Strengths and limitations

The strengths of this study are that it is the largest investigation of police conducted so far, it is nationwide and it represents all occupational levels in the police service. Further, the study applied several validated international instruments. The large number of respondents made multivariate analyses feasible. The comparison with a nationwide cohort of Norwegian physicians is also a strength despite obvious differences between the two groups. Police and physicians are both human service occupations, many of them often working closely with people needing help, making mistakes may be detrimental, they are both dealing with human misery and disasters, etc.

A limitation of the study is the cross-sectional design, which prevents us obtaining direct evidence of causality. Report bias may be a problem, as for example anxiety and depressive symptoms are socially undesirable topics, particularly in a masculine milieu. Comparisons with the general population may be partly misleading because of the healthy worker effect, which reflects that an individual must be relatively healthy in order to be employable in a workforce, and both morbidity and mortality rates within the workforce are usually lower than in the general population [34].

As the samples in the present study are relatively large, some of the differences may be statistically significant, but not necessarily clinically significant.

The external generalizability of the data may also be limited. Policing in Norway differs from that of many other jurisdictions. For example, police are normally unarmed and traditionally the level of crime has been low. On the other hand, there are several similarities between police populations, such as the male-dominated culture and a reluctance to seek help.

## Conclusion

The prevalence of subjective health complaints was relatively high and was mainly associated to job pressure and lack of support. Males showed more depressive symptoms than females. Compared with the general population, though, police showed lower mean scores on both anxiety and depressive symptoms. All stress factors on frequency were positively associated to the burnout dimensions depersonalization and emotional exhaustion, except work injuries. The comparisons with physicians showed that they have markedly different emotional reactions to work stress. Police reported more musculoskeletal pain and scored more highly on depersonalization and all personality dimensions except neuroticism.

## Competing interests

The author(s) declare that they have no competing interests.

## Authors' contributions

AMB was involved in conception and design, acquisition, analysis and interpretation of data and drafting of the manuscript. EH was involved in design, interpretation of data, drafting of the manuscript and supervision. ØE was involved in conception and design, interpretation of data, drafting of the manuscript and supervision. BL was involved in analysis and interpretation of data. AMB is the guarantor for this paper.
